# Penduliflaworosin, a Diterpenoid from *Croton crassifolius*, Exerts Anti-Angiogenic Effect via VEGF Receptor-2 Signaling Pathway

**DOI:** 10.3390/molecules22010126

**Published:** 2017-01-13

**Authors:** Yeyin Liang, Yubo Zhang, Guocai Wang, Yaolan Li, Weihuan Huang

**Affiliations:** 1 Institute of Traditional Chinese Medicine and Natural Products, College of Pharmacy, Jinan University, Guangzhou 510632, China; liangyyangela@163.com (Y.L.); ybzhang99@126.com (Y.Z.); twangguocai@jnu.edu.cn (G.W.); 2 Key Laboratory of Regenerative Medicine, Ministry of Education, Jinan University, Guangzhou 510632, China; 3 Department of Developmental & Regenerative Biology, Jinan University, Guangzhou 510632, China

**Keywords:** penduliflaworosin, anti-angiogenesis, VEGF receptor-2

## Abstract

Anti-angiogenesis targeting vascular endothelial growth factor receptor-2 (VEGFR-2) has been considered as an important strategy for cancer therapy. Penduliflaworosin is a diterpenoid isolated from the plant *Croton crassifolius*. Our previous study showed that this diterpenoid possesses strong anti-angiogenic activity by inhibiting vessel formation in zebrafish. This study was conducted to further investigate the anti-angiogenic activity and mechanism of penduliflaworosin. Results revealed that penduliflaworosin significantly inhibited VEGF-induced angiogenesis processes including proliferation, invasion, migration, and tube formation of human umbilical vein endothelial cells (HUVECs). Moreover, it notably inhibited VEGF-induced sprout formation of aortic rings and blocked VEGF-induced vessel formation in mice. Western blotting studies showed that penduliflaworosin inhibited phosphorylation of the VEGF receptor-2 and its downstream signaling mediators in HUVECs, suggesting that the anti-angiogenic activity was due to an interference with the VEGF/VEGF receptor-2 pathway. In addition, molecular docking simulation indicated that penduliflaworosin could form hydrogen bonds within the ATP-binding region of the VEGF receptor-2 kinase unit. Finally, cytotoxicity assay showed that penduliflaworosin possessed little toxicity toward both cancer and normal cells. Taken together, our findings demonstrate that penduliflaworosin exerts its anti-angiogenic effect via the VEGF receptor-2 signaling pathway. The anti-angiogenic property and low cytotoxicity of penduliflaworosin suggest that it may be useful in cancer treatments.

## 1. Introduction

Angiogenesis is the process by which new blood vessels develop from preexisting vasculature [1]. The angiogenic process plays an important role in tumor growth, invasion, and metastasis because this process increases the supply of nutrients, oxygen, and growth factors to solid tumors and also facilitates the removal of metabolic waste from tumors [2]. Therefore, blocking angiogenesis is an approach to arrest tumor growth and metastasis, and targeting angiogenesis has become an attractive therapeutic strategy for cancer treatment [3,4]. In recent years, angiogenic inhibitors such as bevacizumab, sunitinib, and sorafenib have already been clinically used to treat colorectal cancer, breast cancer, non-small cell lung cancer, and renal cell carcinoma [5,6,7,8].

Angiogenesis is a complex process regulated by a balance between pro- and anti-angiogenic factors. Among these factors, vascular endothelial growth factor (VEGF) is the most potent angiogenesis stimulator [9]. VEGF stimulates endothelial cell proliferation, migration, and tube formation by binding to two major receptor tyrosine kinases (VEGF receptor-1 and VEGF receptor-2) expressed on endothelial cells [10]. Of these two receptors, VEGF receptor-2 mediates the major angiogenic functions of VEGF [10,11]. Activation of VEGF receptor-2 by VEGF induces the phosphorylation of a multitude of proteins in downstream signaling transduction cascades that subsequently promote proliferation, migration, and tube formation of endothelial cells [12]. Those cascades include the extracellular signal-regulated kinase (ERK), the focal adhesion kinase (FAK), the protein kinase B pathway (AKT), and the mammalian target of rapamycin (mTOR) [13]. Thus, VEGF receptor-2 has become an important therapeutic target for the development of anticancer agents [10,14].

**Figure 1:**
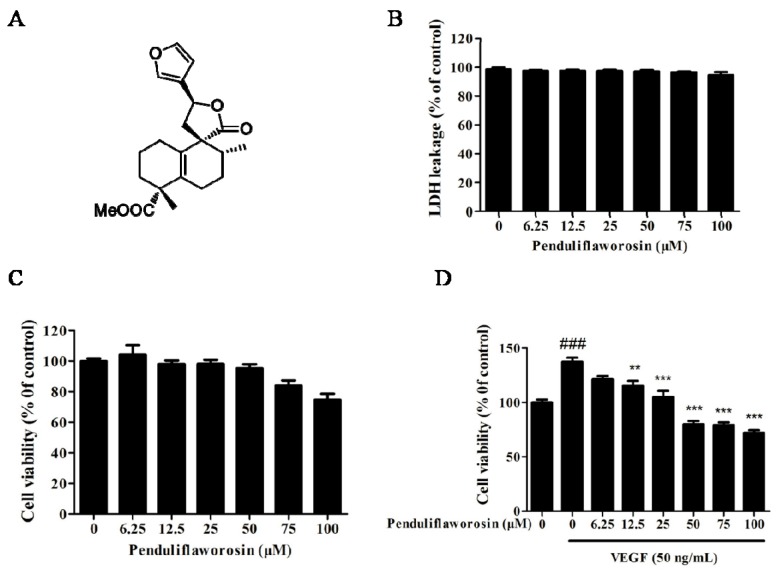
(**A**) The structure of penduliflaworosin. Cytotoxicity of penduliflaworosin in human umbilical vein endothelial cells (HUVECs) was evaluated by (**B**) lactate dehydrogenase (LDH) assay and (**C**) cell-counting assay; (**D**) Penduliflaworosin inhibited vascular endothelial growth factor (VEGF)-induced proliferation of HUVECs in a dose-dependent manner at 48 h. Cells treated with medium only were used as the vehicle control, and data are shown as a percentage of the control. Data are expressed as the mean ± standard deviation (*n* = 3). Mean values showing significant differences between the control group and the VEGF-treated group are indicated by ^###^ (*p* < 0.001). Mean values showing significant difference between the VEGF-treated group and the penduliflaworosin-treated group are denoted by ** (*p* < 0.01) or *** (*p* < 0.001).

The medicinal plant *Croton crassifolius* Geisel (Euphorbiaceae) is widely distributed in southern China and some southern Asian countries. The roots of *C. crassifolius* are used to treat snake bites, stomach ache, sternalgia, joint pain, pharyngitis, jaundice, and rheumatoid arthritis in China [15] and used in cancer treatment in Thailand [16]. Our previous study revealed that diterpenoid derivatives are one of the important anti-angiogenic components of *C. crassifolius* [17,18,19], and penduliflaworosin (Figure 1A) possesses the best activity relative to the positive control in the zebrafish model [19]. In this study, we further explored the anti-angiogenic activity of penduliflaworosin and its molecular mechanism of action.

## 2. Results

### 2.1. Penduliflaworosin Inhibited VEGF-Induced Proliferation of HUVECs

Lactate dehydrogenase (LDH) toxicity assay was carried out to determine whether penduliflaworosin was toxic to the human umbilical vein endothelial cells (HUVECs). As shown in Figure 1B, it had little toxicity on HUVECs at all tested concentrations (0, 6.25, 12.5, 25, 50, 75, and 100 μM). Then, cell-counting assay was used to evaluate its inhibitory effect on HUVEC proliferation. No significant differences in proliferation were observed (*p* > 0.05) at dosages ranging from 0 to 100 μM (Figure 1C), further suggesting little toxicity effect of penduliflaworosin on HUVECs. Next, we studied its inhibitory effect on the proliferation of HUVECs induced by VEGF. When the cells were treated with VEGF for 48 h, the cell number increased to 137.47% of the control (Figure 1D). However, penduliflaworosin significantly suppressed proliferation of VEGF-induced HUVECs in the range of 12.5~100 μM (*p* < 0.05). Overall, penduliflaworosin at noncytotoxic doses significantly inhibited the proliferation of VEGF-stimulated endothelial cells.

### 2.2. Penduliflaworosin Inhibited VEGF-Induced Migration, Invasion, and Tube Formation of HUVECs

**Figure 2:**
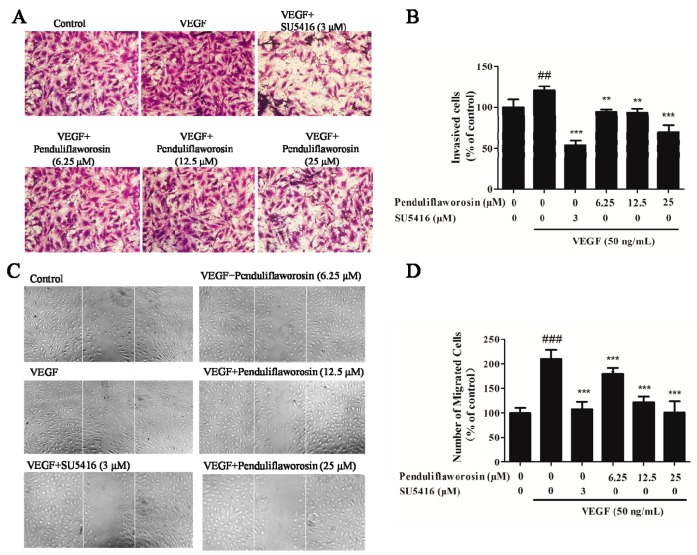
Penduliflaworosin suppressed VEGF-induced invasion and migration of HUVECs. The (**A**) invasion and (**C**) migration of HUVECs were photographed. The numbers of (**B**) invading and (**D**) migrating cells were counted after treatment with compounds (penduliflaworosin or SU5416) at different concentrations. Cells treated with medium only were used as the vehicle control. Data are shown as the percentage of the vehicle-treated control. Values are represented as the mean ± standard deviation (*n* = 3). Mean values showing significant differences between the control group and the VEGF-treated group are denoted by ^##^ (*p* < 0.05) or ^###^ (*p* < 0.001). Mean values showing significant differences between the VEGF-treated group and the penduliflaworosin-treated group are indicated by ** (*p* < 0.01), or *** (*p* < 0.001).

**Figure 3:**
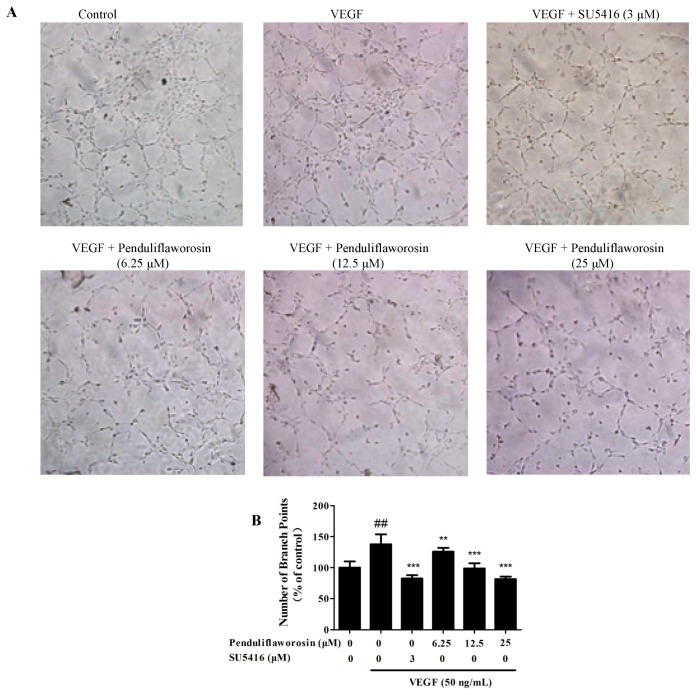
Penduliflaworosin suppressed VEGF-induced tube formation of HUVECs in a dose-dependent manner. (**A**) The tube formation of HUVECs was photographed. The numbers of (**B**) branch points in the tubes were counted after treatment with different concentrations of compounds (penduliflaworosin or SU5416). Cells with medium only were used as the vehicle control, and data are presented as the percentage of the control. Values are represented as the mean ± standard deviation (*n* = 3). Mean values showing significant differences between the control group and the VEGF-treated group are denoted by ^##^ (*p* < 0.001). Mean values showing significant difference between the VEGF-treated group and penduliflaworosin-treated group are indicated by ** (*p* < 0.01) or *** (*p* < 0.001).

Next, we evaluated the potential of penduliflaworosin in inhibiting the migration, invasion, and tube formation of HUVECs induced by VEGF, and SU5416 was used as a positive control [20]. The result showed that a large number of cells migrated across the membrane in the Transwell chamber after stimulation with VEGF, and the number of invasive cells significantly increased to 120.54% of the control. However, in the presence of both penduliflaworosin and VEGF, the number of invasive cells was dramatically reduced in a dose-dependent manner (Figure 2A,B). Additionally, the activity of penduliflaworosin in inhibiting the migration of HUVECs was evaluated by wound-healing assay. As shown in Figure 2C,D, a large number of cells migrated to the gap after stimulation only with VEGF. When the cells were treated with both penduliflaworosin and VEGF, the number of migrating cells was dramatically decreased in a dose-dependent manner. The result indicated that penduliflaworosin could weaken the migration capability of HUVECs. Similarly, VEGF-induced HUVECs showed well-formed tubular structures in the absence of penduliflaworosin (Figure 3). However, when the VEGF-treated cells were treated with increasing concentrations of penduliflaworosin, tube formation was gradually interrupted. Taken together, the results showed that penduliflaworosin inhibited the migratory process and tube formation of HUVECs induced by VEGF.

### 2.3. Penduliflaworosin Inhibited VEGF-Induced Vessel Sprout Formation

**Figure 4:**
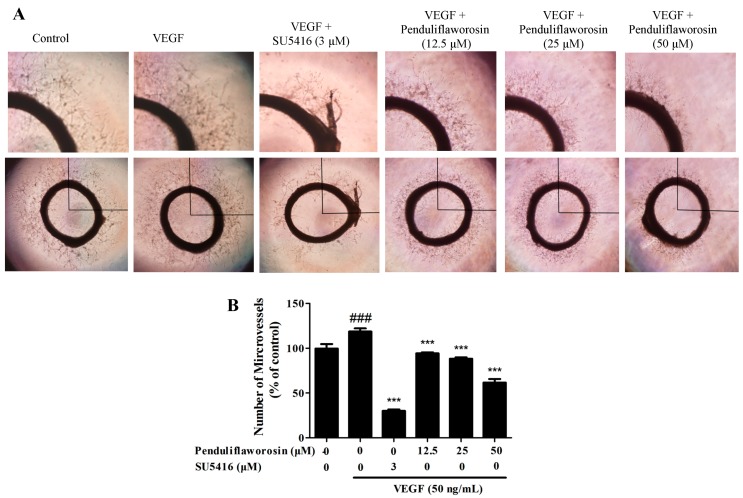
Penduliflaworosin inhibited VEGF-induced vessel sprout formation in a dose-dependent manner. Aortic rings isolated from Sprague-Dawley rats were cultured in wells pre-coated with Matrigel and treated with different concentrations of compounds (penduliflaworosin or SU5416) and VEGF (50 ng/mL) for 4 days. Aortic rings treated with culture medium only were used as the vehicle control. (**A**) The sprouting of microvessels from the aortic rings was photographed; (**B**) The numbers of microvessels sprouting from aortic rings were counted after treatment with the compounds. Aortic rings treated with medium only were used as the vehicle control, and data are shown as a percentage of the control. Values are represented as the mean ± standard deviation (*n* = 3). Mean values showing significant differences between the control group and the VEGF-treated group are denoted by ^###^ (*p* < 0.001). Mean values showing significant difference between the VEGF-treated group and penduliflaworosin-treated group are indicated by *** (*p* < 0.001).

The in vitro experiments showed that penduliflaworosin could inhibit angiogenesis. To further determine whether it inhibited angiogenesis ex vivo, we conducted organotypic assay using a rat aortic arch model and measured endothelial cell outgrowth. As shown in Figure 4, compared to the control, sprouts around the ring treated with VEGF were longer, and more cells migrated into the matrix. When the rings were treated with both VEGF and increasing concentrations of penduliflaworosin, the sprouts around the rings were shorter, suggesting that penduliflaworosin could inhibit the sprout length and density after stimulation with VEGF.

### 2.4. Penduliflaworosin Inhibited VEGF-Induced Blood Vessel Formation in Mice

**Figure 5:**
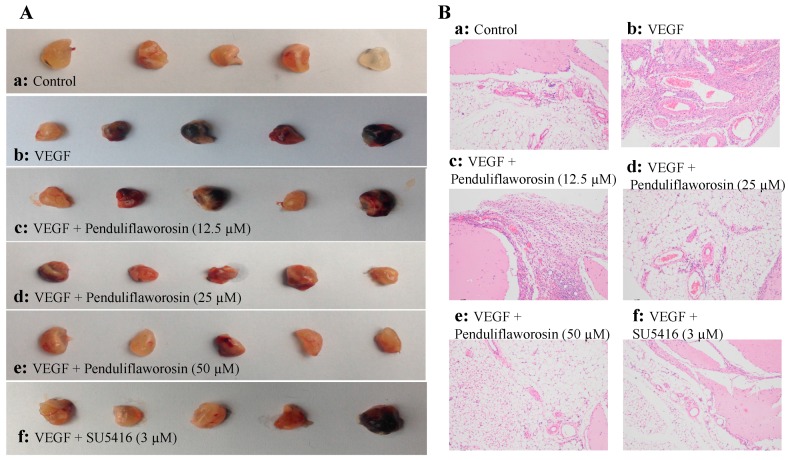
Penduliflaworosin inhibited VEGF-induced blood vessel formation in vivo. The ventral area of C57/BL/6 mice was injected with 300 µL of Matrigel containing various concentrations of compounds in presence of VEGF (250 ng) and heparin (150 units). After incubation for 21 days, the Matrigel plugs were harvested. (**A**) The Matrigel plugs were photographed; (**B**) the Matrigel plugs were fixed, sectioned, and stained with hematoxylin and eosin (magnification, ×200).

We further assessed the anti-angiogenic effect of penduliflaworosin in vivo. As shown in Figure 5A, the Matrigel plugs containing VEGF only were dark red in color and filled with blood vessels, suggesting that functional vasculature had formed. However, the Matrigel plugs with both VEGF and increasing concentrations of penduliflaworosin (12.5, 25, and 50 μM) were lighter in color, suggesting that fewer blood vessels were formed. Furthermore, after staining with hematoxylin and eosin, fewer vessels were observed in the penduliflaworosin-treated plugs (Figure 5B). The result showed that penduliflaworosin inhibited VEGF-induced blood vessel formation in vivo.

### 2.5. Penduliflaworosin Attenuated VEGF Receptor-2 Tyrosine Kinase Activity and the VEGF Receptor-2 Signaling Pathway

**Figure 6:**
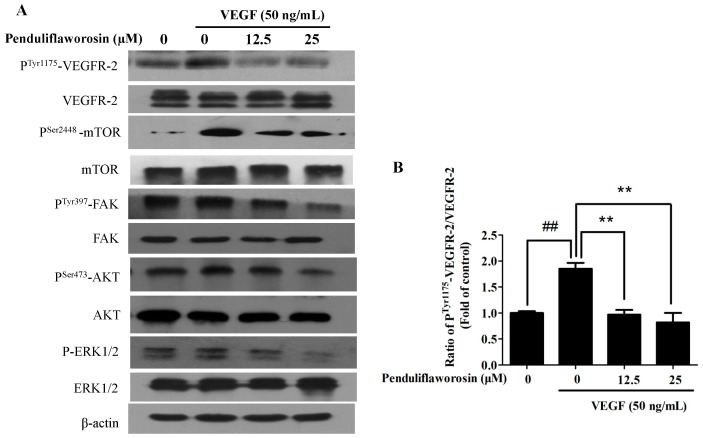

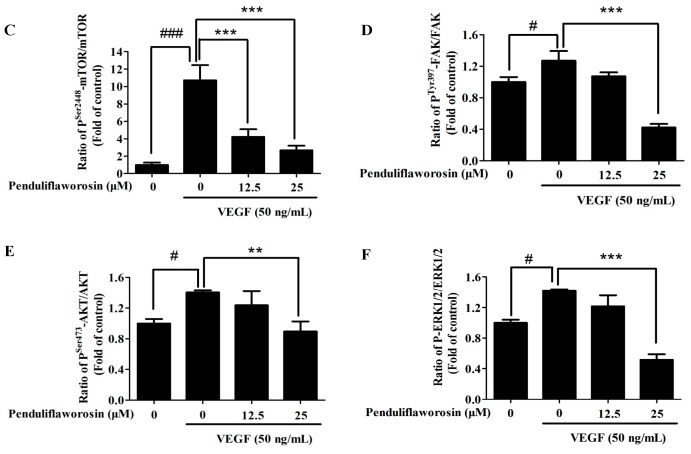
(**A**) Effect of penduliflaworosin on the expression of the phosphorylation of VEGF receptor-2 (VEGFR-2) and VEGFR-2 downstream signaling molecules, including mammalian target of rapamycin (mTOR), focal adhesion kinase (FAK), protein kinase B (AKT), and extracellular signal-regulated kinase (ERK) in HUVECs in a dose-dependent manner. (**B**–**F**) The ratios of phospho^Tyr1175^-VEGFR-2/VEGFR-2, phospho^Ser2448^-mTOR/mTOR, phospho^Tyr397^-FAK/FAK, phospho^Ser473^-AKT/AKT, and phospho-ERK1/2/ERK1/2 were determined, respectively. Values are represented as the mean ± standard deviation (*n* = 3). Mean values showing significant differences between the control group and the VEGF-treated group are denoted by ^#^ (*p* < 0.05), ^##^ (*p* < 0.01), or ^###^ (*p* < 0.001). Mean values showing significant difference between the VEGF-treated group and penduliflaworosin-treated group are indicated by ** (*p* < 0.01) or *** (*p* < 0.001).

The above results suggested that penduliflaworosin effectively inhibited VEGF-induced angiogenesis both in vitro and in vivo. We then examined its effect on tyrosine phosphorylation of VEGF receptor-2 (p-VEGFR-2, the active form of VEGF receptor-2) stimulated by VEGF. As shown in Figure 6, when the cells were treated with penduliflaworosin, the expression of p-VEGFR-2 (Tyr1175) was reduced in a dose-dependent manner. Meanwhile, the total levels of VEGF receptor-2 were not significantly altered. The results showed that penduliflaworosin affected the interaction between VEGF and VEGF receptor-2. In addition, phosphorylation of VEGF receptor-2 subsequently triggers multiple downstream signals to induce proliferation and differentiation of endothelial cells [21], including AKT, mTOR, FAK, and ERK [22,23]. The results of Western blotting assay showed that penduliflaworosin inhibited VEGF-stimulated phosphorylation of AKT, mTOR, FAK, and ERK (p-AKT, p-mTOR, p-FAK, and p-ERK), but the total levels of AKT, mTOR, FAK, and ERK were almost unaffected.

### 2.6. Penduliflaworosin Bound the ATP-Binding Sites of the VEGFR-2 Kinase Domain

**Figure 7:**
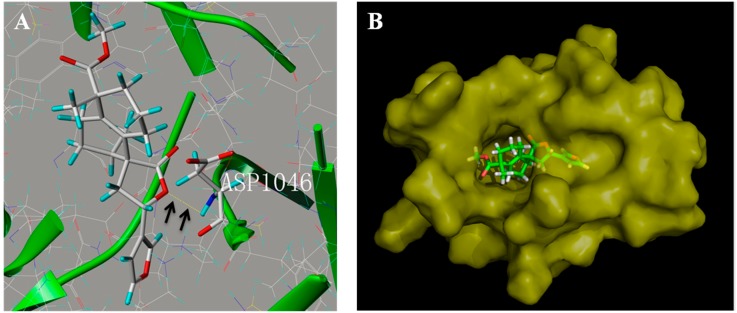
Penduliflaworosin interacted with the ATP-binding sites of VEGF receptor-2 kinase domain. (**A**) Penduliflaworosin formed hydrogen bonds with residue Asp1046. (**B**) MOLCAD surface modeling showed that the furan ring of penduliflaworosin extended into the deep cavity of the ATP-binding pocket of VEGF receptor-2.

We next analyzed the binding between penduliflaworosin and the VEGF receptor-2 kinase domain to further elucidate how penduliflaworosin exerts its anti-angiogenic effect via VEGF receptor-2. For this purpose, computer-docking simulations of the interaction of VEGF receptor-2 with penduliflaworosin were carried out. As shown in Figure 7A, the O motif at the lactonic ring of penduliflaworosin formed a hydrogen bond with the backbone NH of Asp1046, which suggested that residue Asp1046 may be the ligand-binding site between penduliflaworosin and VEGF receptor-2. Moreover, MOLCAD surface modeling showed that the furan ring of penduliflaworosin extended into the deep cavity of the ATP-binding pocket of VEGFR-2 (Figure 7B). The binding mechanism of penduliflaworosin to VEGF receptor-2 may provide structural insight for the development of small natural inhibitors.

## 3. Discussion

Penduliflaworosin is a clerodane-type diterpenoid isolated from *Croton crassifoliu*s (Euphorbiaceae). It has also been found in other *Croton* species (*Croton penduliflorus* and *Croton jatrophoides*) in recent years [24,25]. However, its bioactivity has rarely been studied. Our previous study examined its anti-angiogenic activity preliminarily using a zebrafish model. It is believed that blocking the VEGF/VEGF receptor-2 pathway can effectively inhibit tumor angiogenesis, metastasis, and leakage [10]. The results of this study showed that penduliflaworosin could inhibit VEGF-induced angiogenesis both in vitro and in vivo. Penduliflaworosin inhibited several important steps of angiogenesis in VEGF-stimulated HUVECs, VEGF-stimulated sprout formation of the rat aortic arch, and VEGF-stimulated blood vessel formation in mice.

Then, the intrinsic mechanisms underlying the inhibition of VEGF-induced angiogenic activity by penduliflaworosin were investigated. As the activity of VEGF is predominantly mediated by its high affinity with the endothelial cell receptor (VEGF receptor-2), inhibition of VEGF receptor-2 has been proposed as a strategy for therapeutic intervention. VEGF receptor-2 undergoes auto-phosphorylation principally at Tyr1175 sites in its intracellular kinase domain and then initiates a series of downstream signal transductions in endothelial cells [21]. In the present study, penduliflaworosin inhibited VEGF-induced tyrosine phosphorylation of VEGF receptor-2 and its downstream signals, including AKT, mTOR, FAK, and ERK. The AKT/mTOR pathway is critical for control of cell survival and differentiation in various cell types, such as endothelial cells, human breast cancer epithelial cells, and smooth muscle cells [23]. ERK and FAK play important roles in the progression of different cancers and are important signaling effectors linked to cell adhesion, invasion, proliferation, migration, and survival in many cancers [22]. These results showed that penduliflaworosin suppressed the VEGF receptor-2 pathway to inhibit angiogenesis. Meanwhile, the in vitro findings were also consistent with our in vivo results, indicating that the inhibition of blood vessel formation in mice may be due to the inhibitory effect of penduliflaworosin on phosphorylated VEGF receptor-2 and its downstream signaling molecules.

Moreover, computational docking experiment showed that penduliflaworosin occupied a deep hydrophobic pocket in the ATP-binding site of VEGF receptor-2 by forming a hydrogen bond. The O motif at the lactonic ring of penduliflaworosin stabilized its occupation of the hydrophobic pocket of VEGF receptor-2. These data further suggested that penduliflaworosin inhibits angiogenesis by targeting VEGF receptor-2.

Notably, we found that penduliflaworosin possessed little cytotoxicity to HUVECs at the tested concentrations based on LDH assays, indicating that its anti-angiogenic effect was not likely due to toxicity at the cellular level. Toxicity towards cancer cells to induce cell death is a common anticancer therapeutic strategy [26]. Therefore, the cytotoxic effect of penduliflaworosin on cancer cells—including human larynx epidermoid carcinoma cells (HEp-2), human hepatocellular liver carcinoma cells (HepG2), human breast carcinoma (MCF7), and human nasopharyngeal cancer cells (CNE)—was further investigated. The results showed that there was little cytotoxicity towards the cancer cells at all concentrations evaluated (12.5–100 μM) (data were not shown). In fact, penduliflaworosin showed little cytotoxicity towards both cancer cells and normal cells. Although the anti-angiogenic activity of penduliflaworosin is lower than that of the available compound (SU5416), it has little cytotoxicity. These results provide an experimental basis for further study of penduliflaworosin as a safe anticancer compound. 

Overall, our study indicated that penduliflaworosin at nontoxic dosages exerted potent anti-angiogenic activity by specifically targeting the VEGF receptor-2 signaling pathway. Our findings may offer new insights for the application of penduliflaworosin in anti-angiogenic therapy.

## 4. Materials and Methods

Penduliflaworosin was previously isolated from the roots of *C. crassifolius*, and the compound had >98% purity as determined by high-performance liquid chromatography [27].

### 4.1. Cell Culture

HUVECs were purchased from the American Type Culture Collection (Manassas, VA, USA) and maintained in Dulbecco’s modified Eagle’s medium with nutrient mixture F-12 (DMEM/F12) supplemented with 20% fetal bovine serum (FBS, Gibco, Gaithersburg, MD, USA), heparin (0.1 mg/mL, Sigma, Santa Clara, MA, USA), basic fibroblast growth factor (5 ng/mL, PeproTech, Rocky Hill, NJ, USA) and epidermal growth factor (10 ng/mL, Invitrogen, Carlsbad, CA, USA). HUVECs at early passages (passages 3–7) were used in all the experiments. HEp-2, HepG2, MCF7, and CNE cells were obtained from the American Type Culture Collection (ATCC, Manassas, VA, USA). Eagle’s minimum essential medium (EMEM, Rockville, MD, USA) with 10% FBS was used to culture the cancer cells. All cells were incubated at 37 °C with 5% CO_2_.

### 4.2. Animals

Sprague-Dawley rats (male, 220–240 g) and C57/BL/6 mice (male, 6 weeks old) were purchased from the Guangdong Medical Laboratory Animal Center (Guangzhou, China). The animals were kept in an environmentally controlled room (12 h dark/light cycle, temperature: 25 ± 1 °C, relative humidity: 50% ± 5%). All the animal experiments were conducted under the supervision and assessment by Laboratory Animal Ethics Committee Jinan University.

### 4.3. LDH Toxicity Assay

Cells were seeded in 96-well plates (6000 cells per well) for overnight attachment. The growth medium (DMEM/F12 containing 20% FBS for HUVECs; EMEM containing 10% FBS for cancer cells) was discarded. The cells were further incubated in fresh medium containing various compounds at different concentrations for 48 h. After treatment, the cell supernatants were collected and analyzed for LDH activity using a Cytotoxicity Detection Kit (Roche Diagnostics GmbH, Mannheim, Germany) according to the manufacturer’s instructions.

### 4.4. Cell-Counting Assay

The inhibitory effect of compound on the viability of HUVECs was measured using a cell-counting assay (cell counting kit (CCK)-8, Dojindo, Kumamoto, Japan) according to the method of Huang et al. [28]. Briefly, HUVECs (6000 cells per well) were seeded in a 96-well plate for overnight attachment. The growth medium was replaced with fresh medium containing various concentrations of compounds. After incubation for 48 h, the medium was replaced with DMEM/F12 containing 10% 2-(2-methoxy-4-nitrophenyl)-3-(4-nitrophenyl)-5-(2,4-disulfophenyl)-2*H*-tetrazolium sodium salt (WST-8). After being further incubated for 4 h, the absorbance was measured at 450 nm with a microplate reader (Thermo Fisher Scientific, New York, NY, USA).

The inhibitory effect of compounds on the viability of VEGF-induced cells viability was also measured using the cell-counting assay. HUVECs (6000 cells per well) were seeded in a 96-well plate. After incubation with 50 ng/mL of VEGF (VEGF-A165, PeproTech, Jersey City NJ, USA) in the presence or absence of various concentrations of compounds for 48 h, the medium was replaced with DMEM/F12 containing 10% WST-8. After being further incubated for 4 h, the absorbance was measured at 450 nm with a microplate reader. The cells incubated only with medium were set as a vehicle control. 

### 4.5. Wound-Healing Assay

HUVECs were cultivated in 48-well plates (2.5 × 10^4^/well) and allowed to grow to 90% confluence. First, the cells were incubated with DMEM/F12 (200 μL/well) for 6 h. A scrape was made in the middle of the well by a 10 μL tip followed by washing with phosphate-buffered saline (PBS) twice, and then the cells were incubated with growth medium containing VEGF (50 ng/mL) with different concentrations of compounds. After 8 h of treatment, the cells were photographed, and the migrated cells were calculated. Cells incubated with growth medium only were used as the vehicle control. The effect of the compounds on migration was evaluated by comparing the number of migrated cells of the compound-treated groups to that of the control group [29].

### 4.6. Invasion Assay

The Transwell system was applied to perform the invasion assay [29]. Transwells (8 μm pore; Corning, New York, NY, USA) were pre-coated with Matrigel (BD Bioscience Company, Franklin Lakes, NJ, USA). The upper chambers were filled with HUVECs (5 × 10^4^ cells per chamber) in DMEM/F12 with 1% FBS (100 μL), while the bottom chambers were filled with the same medium (600 μL) containing VEGF (50 ng/mL). The upper chambers contained different concentrations of compounds. After incubation for 24 h, the invaded cells were fixed and stained. Cells incubated with the medium only were used as the vehicle control. Finally, the invaded cells were photographed and counted.

### 4.7. Tube Formation Assay

The HUVECs (4~5 × 10^4^ cells/well) were seeded in the wells pre-coated with Matrigel in 48-well culture plates, and growth medium (200 μL/well) containing VEGF (50 ng/mL) in the presence or absence of different concentrations of compounds was added. After 8 h incubation, the tubular structures were photographed, and the number of tubular structures was calculated. Cells treated with growth medium only were used as the vehicle control [29].

### 4.8. Aortic Ring Assay

A 96-well culture plate was pre-coated with Matrigel (50 μL/well) for 30 min. The aortic rings (1–1.5 mm long) isolated from Sprague-Dawley rats were placed in the wells pre-coated with Matrigel and covered with Matrigel (70 μL/well). They were then incubated at 37 °C in growth medium containing VEGF (50 ng/mL) in the presence or absence of compounds at various concentrations for 4 days. Finally, microvessel growth was observed, and the number of branching sites was calculated. Rings treated with growth medium only were used as the vehicle control [28].

### 4.9. Matrigel Plug Assay

Thirty C57/BL/6 mice were used in the Matrigel plug assay as described previously [28]. The growth medium (250 μL) containing VEGF (250 ng), heparin (150 units), and various concentrations of compounds was added to Matrigel (250 μL per plug) and then mixed well. The mixture was injected into the ventral area of the mice (five mice per group). After 21 days of incubation in vivo, the Matrigel plugs were removed, fixed, and then cut into sections using the paraffin sectioning method and stained by hematoxylin and eosin staining. Matrigel containing neither VEGF nor compounds was used as the vehicle control.

### 4.10. Western Blotting

HUVECs were incubated for 4 h in DMEM/F12 containing 4% FBS with various concentrations of compound after 6 h of serum starvation. Then, the medium was replaced with DMEM/F12 containing 4% FBS and VEGF (50 ng/mL), and further incubated for 1 h. Cell lysates were separated by 8% SDS-PAGE and transferred to polyvinylidene difluoride membranes. Membranes were incubated with primary antibodies (Cell Signaling Technology, Danvers, MA, USA) at 4 °C overnight. After incubation, membranes were washed and incubated with secondary antibodies (Cell Signaling Technology, Danvers, MA, USA) for 2 h at room temperature. Immunoreactive bands were then visualized by the enhanced chemiluminescence detection system [28].

### 4.11. Molecular Docking

The molecular docking study was carried out using the molecular modeling software package SYBYL 8.0 (Tripos, St. Louis, MO, USA). The crystal structure of VEGF receptor-2 was identified from the Protein Data Bank (http://www.rcsb.org/pdb/home/home.do), and then, file 3VHK.pdb was downloaded. First, the crystallographic ligand was extracted from the active site, and the residues within a 6.5 Å radius around the VEGFR-2 molecule were defined as the active site. Then, the molecular structure was charged and underwent energy minimization. The Surflex-Dock program (version 8.0, Tripos, St. Louis, MO, USA) was used for the docking calculations with default parameters. Finally, the protein–ligand complex was obtained, and the MOLCAD surfaces were generated for visualizing the binding mode of the docked protein–ligand complexes.

### 4.12. Statistical Analysis

Data are shown as the mean ± standard deviation. Statistical analysis was performed using one-way ANOVAs followed by Dunnett’s test, with *p* = 0.05 defined as statistically significant.
